# Micro- and Nanosized Substances Cause Different Autophagy-Related Responses

**DOI:** 10.3390/ijms22094787

**Published:** 2021-04-30

**Authors:** Yung-Li Wang, Cai-Mei Zheng, Yu-Hsuan Lee, Ya-Yun Cheng, Yuh-Feng Lin, Hui-Wen Chiu

**Affiliations:** 1 Graduate Institute of Clinical Medicine, College of Medicine, Taipei Medical University, Taipei 11031, Taiwan; cetuspower@gmail.com (Y.-L.W.); linyf@s.tmu.edu.tw (Y.-F.L.); 2 Division of Nephrology, Department of Internal Medicine, Shuang Ho Hospital, Taipei Medical University, New Taipei City 23561, Taiwan; 11044@s.tmu.edu.tw; 3 TMU Research Center of Urology and Kidney, Taipei Medical University, Taipei 11031, Taiwan; 4 Department of Internal Medicine, School of Medicine, College of Medicine, Taipei Medical University, Taipei 11031, Taiwan; 5 Department of Cosmeceutics, China Medical University, Taichung 406040, Taiwan; yhlee@mail.cmu.edu.tw; 6 Department of Environmental Health, Harvard University T.H. Chan School of Public Health, Boston, MA 02115, USA; yayun_cheng@hsph.harvard.edu; 7 Department of Medical Research, Shuang Ho Hospital, Taipei Medical University, New Taipei City 23561, Taiwan

**Keywords:** microparticles, nanoparticles, autophagy, extracellular vesicles

## Abstract

With rapid industrialization, humans produce an increasing number of products. The composition of these products is usually decomposed. However, some substances are not easily broken down and gradually become environmental pollutants. In addition, these substances may cause bioaccumulation, since the substances can be fragmented into micro- and nanoparticles. These particles or their interactions with other toxic matter circulate in humans via the food chain or air. Whether these micro- and nanoparticles interfere with extracellular vesicles (EVs) due to their similar sizes is unclear. Micro- and nanoparticles (MSs and NSs) induce several cell responses and are engulfed by cells depending on their size, for example, particulate matter with a diameter ≤2.5 μm (PM2.5). Autophagy is a mechanism by which pathogens are destroyed in cells. Some artificial materials are not easily decomposed in organisms. How do these cells or tissues respond? In addition, autophagy operates through two pathways (increasing cell death or cell survival) in tumorigenesis. Many MSs and NSs have been found that induce autophagy in various cells and tissues. As a result, this review focuses on how these particles interfere with cells and tissues. Here, we review MSs, NSs, and PM2.5, which result in different autophagy-related responses in various tissues or cells.

## 1. Introduction

Micro- and nanomaterials with different physical and chemical properties have been developed for human needs [1,2]. However, micro- and nanomaterials also show unexpected toxicity [3]. Nanotoxicology is rapidly developing with potential hazardous effects for nanomaterials [4,5]. Due to their larger sizes and small surface-to-volume ratios, micromaterials are considered less toxic than nanomaterials. In addition, nanomaterials can aggregate to a microscale size [6,7]. Micromaterials are also harmful to humans [3,8]. These materials may return to humans via the food chain [9,10]. Human consumption of micro- or nanoplastics may occur through seafood [11,12], water [13,14], etc. However, PM2.5 (particulate matter ≤ 2.5 μm) is a mix of micro- and nanosized substances (MSs and NSs) that can cause many chronic diseases [15,16]. Many studies show that MSs and NSs are toxic [17]. These related materials have a potential risk to human health.

Air pollution has become increasingly severe due to the process of industrialization and urbanization in many developing countries [18,19]. Particulate matter (PM) is composed of different sized solids and/or liquids suspended in the air. According to their diameter, PM can be grouped into the following four different categories: total suspended particulates; PM with a diameter <10 μm (PM10); fine PM with a diameter < 2.5 μm (PM2.5); and ultrafine PM with a diameter < 0.1 μm (PM0.1) [20,21]. When people breaths, PM2.5 is easily inhaled, is deposited in the airways and alveoli, penetrates the blood, circulates, and induces damage to tissues and organs [22,23]. PM2.5 is mainly derived from industrial factories, petroleum-consuming vehicle emissions, dust from mining, combustion, etc. In summary, the composition of PM2.5 is overly complicated [24,25,26,27]. PM2.5 particles can interact with metals, inorganic particles, organic carbon, and microbes, resulting in toxic reactions, such as inflammation, DNA damage, reactive oxygen species (ROS) induction, and mitochondria dysfunction. These unusual responses in cells are related to the pathogenesis of a series of human diseases [28,29,30], such as emphysema in mice [31], lung cancer, and chronic airway inflammatory diseases [32]; the promotion of cancer stem cell properties [33]; the impairment sperm quality in mice [34]; the induction of allergic airway inflammation in mice [35]; and the development of Alzheimer’s disease [36], PM2.5 induces not only oxidative stress but also autophagy [37,38].

**Figure 1:**
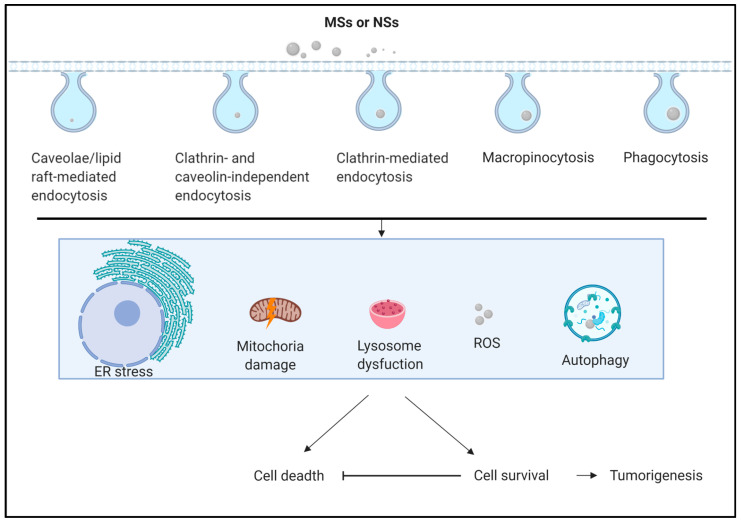
Schematic picture of several major endocytosis pathways for micro- and nanosized substances (MSs and NSs). MSs and NSs employ one or multiple endocytosis pathways to enter cells. The main endocytosis pathways of MSs or NSs include clathrin-mediated endocytosis, caveolae/lipid raft-mediated endocytosis, clathrin- and caveolin-independent endocytosis, macropinocytosis and phagocytosis. The possible mechanisms by which MSs and NSs modulate several cell responses, such as ER-stress, mitochondrial damage, lysosome dysfunction, ROS production, and autophagy, are summarized. MSs: Micro-sized substances; NSs: Nanosized substances.

**Figure 2:**
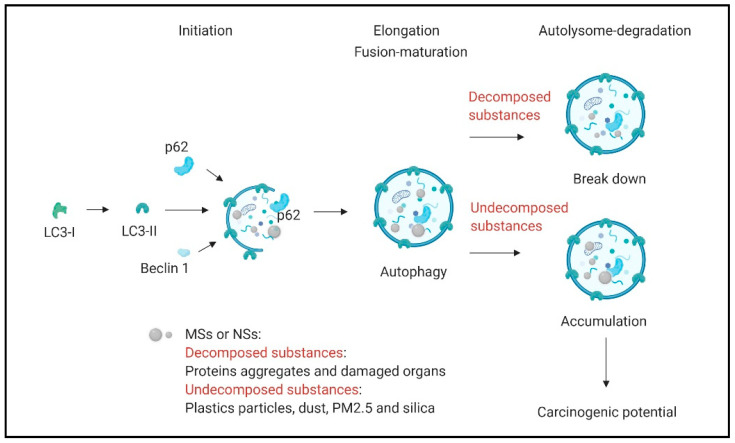
Schematic picture of the macroautophagy process. MNs and NSs, including protein aggregates, damaged organs, plastics particles, dust, and silica are shown. LC3-II, Beclin 1, and p62 conjugate enzymes generate the phagophore form and then the surrounding MNs and NSs during the elongation stage. At the end of the elongation stage, the membrane is sealed to form a double-membrane vesicle, called the autophagosome, which contains degraded cellular enzymes. The autophagosome fuses with a lysosome, forming an autolysosome in which lysosomal enzymes degrade the cargo and release the degraded products into the cytoplasm. Undecomposed MSs and NSs, such as dust and silica, have carcinogenic potential.

MSs and NSs use several endocytosis pathways to enter cells [39,40]. The main endocytosis pathways include clathrin-mediated endocytosis [41], caveolae/lipid raft-mediated endocytosis [42], clathrin- and caveolin-independent endocytosis [43], macropinocytosis [44] and phagocytosis [45] (Figure 1). In a previous report, endocytosis was found to be associated with autophagy [46]. MSs and NSs can also be degraded by a lysosome after entering the cells through three main pathways, including macroautophagy, microautophagy, chaperone-mediated autophagy (CMA), and ribonucleic/deoxyribonucleic (RN/DN)-autophagy [46,47,48,49]. Microautophagy refers to the process by which cells directly take up a substance by invagination or scission from the lysosomal membrane [50,51] (Figure 2). CMA differs from microautophagy in that it does not use membranous structures to separate cargo. CMA uses chaperones to identify cargo proteins that contain a specific peptide motif [52,53]. Macroautophagy, which refers to common autophagy, is the process responsible for the degradation of large cargoes, such as damaged organelles, intracellular pathogens, and protein aggregates. In macroautophagy, a double membrane structure protrudes from the endoplasmic reticulum (ER) and, extends into a ball-like structure after its closure into an autophagosome [54,55]. Autophagy plays an important role in most eukaryotes and is highly conserves in the cellular process of many mammalian on autophagy-related genes (Atgs) [56]. The primary processes regulate autophagy as the phagophore assembly, autophagosome formation and maturation and autolysosome degradation [57]. In addition, autophagy can interfere with the regulation of core metabolism [58,59], damage control [60,61], and cell death [62,63]. Many proteins regulate autophagy, such as unc-51-like autophagy activating kinase 1 (ULK1), Atg13, focal adhesion kinase (FAK) family kinase-interacting protein 200 (FIP200), phosphatidylinositol 3-phosphate (PI3P), vacuolar protein sorting-associated protein 34 (Vps34), Vps15, Beclin1, and Atg14 in the phagophore assembly. In addition, Atg12, Atg10, Atg5, p62, Atg4, and LC3 participate in other autophagy processes [57]. The autophagosome fuses with a lysosome to an autolysosome that contains over 60 hydrolases [64,65], such as cathepsin B, cathepsin L, legumain [66], and cathepsin D [67]. If some materials in the autolysosome are not easily decomposed in the autophagy process, how do these cells or tissues respond? There are several diseases, such as pneumoconiosis, silicosis, and asbestosis caused by silica or undecomposed substances [68]. It is unclear whether previous studies have provided solutions for preventing the effects of micro- and nanosized substances and PM2.5.

Extracellular vesicles (EVs) are defined as lipid-bound particles of various sizes secreted from cells to extracellular spaces or circulated to target tissues [69,70]. EVs can be briefly classify into three types based on their size and biogenesis [71,72]. Small EVs are 50–100 nm in size and, include exosomes and endosome-derived membrane vesicles that are formed from multivesicular bodies (MVBs), intraluminal vesicles (ILVs) and the cellular plasma membrane [73,74]. Microvesicles (MVs), microparticles (MPs) and ectosomes are considered large EVs that are shed directly from the cell surface [73,75]; apoptotic bodies are formed during apoptosis genesis, and their diameters range between 1000 and 5000 nm [76,77]. Previous studies have shown that EVs have important biological relevance, such as immunity and inflammation [78,79], hemostasis [80,81], reproduction [82], and tumorigenesis [83]. Recently, conditioned medium from stem cells is as a new therapeutic application [84,85]. Conditioned medium applicates in diabetic wound healing [86,87], preventing activation of keloid fibroblasts in human [88], musculoskeletal tissue regeneration [89], hair regeneration in human [90], retinal ischemia-reperfusion in rat [91], differentiation of rat retinal progenitor cells [92], promoting survival and neurite outgrowth of neural stem cells in canine [93], autoimmune encephalomyelitis in mice [94], spinal cord injury in canine [95], lung injury and disease [96], EVs can isolated form conditioned medium [97]. Therefore, EVs have sizes similar to those of MSs and NSs or particulate matter less than 2.5 μm (PM2.5). Whether these micro- and nanoparticles interfere with the function of EVs is still unclear.

## 2. Classification of Micro- and Nanosized Substances (MSs and NSs)

Microparticles and nanoparticles are particulate particles with a size ranging from 1–1000 μm or 1–1000 nm, respectively [98]. The sources of MSs and NSs can be classified into three main categories based on their origin. There are three main categories (A), (B), and (C).

(A) Unexpected MSs and NSs are produced through industrial processes, such as particles produced from urban dust, non-exhaust vehicle emissions, vehicle engine exhaust, road dust, welding fumes, combustion processes and even some natural processes, such as forest fires or volcano bursts [99]. Automobile exhaust or diesel engines release approximately 20–130 nm sized particles. Therefore, gasoline engines release approximately 20–60 nm sized particles [100,101]. In addition, plastics originate from synthetic polymers produced by the polymerization of monomers [102]. Plastic is divided into polyamides (PA), polycarbonate (PC), polyethylene (PE), polyester (PES), polyethylene terephthalate (PET), polyetherimide (PEI), polystyrene (PS), polypropylene (PP), polyvinyl chloride (PVC), polyvinylidene chloride (PVDC), low-density polyethylene (LDPE), high-density polyethylene (HDPE), high impact polystyrene (HIPS), acrylonitrile butadiene styrene (ABS), polycarbonate/acrylonitrile butadiene styrene (PC/ABS), polyurethanes (PU), polymethyl methacrylate (PMMA), polytetrafluoroethylene (PTFE) melamine formaldehyde (MF), and urea–formaldehyde (UF) [103]. These plastics can be fragmented into smaller pieces by ultraviolet light and biodegraded [104]. Furthermore, the mixed synthetic fibers textiles we wear daily also generate MSs or NSs from laundering [105,106,107,108]. Briefly, synthetic fibers contain glass and ceramic fibers, aramid fibers, viscose rayon fibers, carbon fibers, polyolefin fibers, nylon fibers [108]. Synthetic fiber textiles have been found to produce microfibers from PES, PE, PP, LDPE, HDPE, PA, and rayon in marine sediments [109]. A single garment may release over 1900 fibers per washing [110]. On the other hand, car tires are an abundant source of microplastics in the environment [111]. Tire wear particles have been studied and calculated in many regions, such as the USA [112], China [113], France, Japan [114], among other countries [115]. A previous study indicated that the main mass of tire wear particle is over 100 μm [116]. In addition, airborne vehicle-derived Fe-bearing nanoparticles also flow into the environment [116]. Artificial turf, brake wear, airplane tires, and road markings also contribute microplastics to the environment [111,115]. Non-exhaust vehicular emissions are caused by road dust, which is itself generated by tire, brake, and clutch wear; road surface wear, and the degradation of other vehicles and road markings [117]. Furthermore, cigarette smoke and building demolition also produce MSs or NSs. Cigarette smoke produces NSs ranging from 10–700 nm [118].

(B) Engineered MSs and NSs have been manufactured by humans to possess certain properties required for their desired applications [119,120]. There are serval types of nanomaterials. For examples, inorganic nanomaterials contain iron oxide nanomaterials, gold nanomaterials, silver nanomaterials, carbon-based nanomaterials, silica nanomaterials, zinc oxide nanomaterials, quantum dots, rare earth oxide nanomaterials, alumina nanomaterials, titanium dioxide nanomaterials [121], and copper oxide nanoparticle [122]. Carbon-based nanomaterials also contain fullerenes, carbon nanotubes, carbon nanofibers, carbon black, carbon onions, and graphene [123]. In addition, dendrimers, micelles, and liposomes belong to engineered NSs [123]. Furthermore, electronic and photovoltaic devices often use gallium- and indium-based oxide and arsenide nanoparticles [124]. Besides, the NS wastewater from semiconductor manufacturing has the potential to enter the ecosystem [125].

(C) Naturally produced MSs and NSs can be found in organisms, such as viruses, bacteria, worms, insects, plants, animals and humans [123,126,127,128,129]. MSs and NSs significantly increase with industrial processes [27,123], human usage [105,130] and garbage fragments [131]. With the discovery of new techniques, more MSs or NSs contaminations have been found, making biosafety a challenging issue [132]. Besides, these MSs or NSs can accumulate in our food chain. Humans can potentially accumulate MSs and/or NSs in the gut, the liver, the kidney [133,134], and muscle tissue [135], as well as from food items, such as fish [136], seafood [11,137,138], milk [132,139], beer [140,141], sea salt [141,142], sugar [143], honey [143], plastic teabags [144], raw water [145], tap water [141], and bottled water [146,147]. In particular, some MSs and NSs engulfing cells are not easily decomposed.

## 3. Autophagy-Related Responses to MSs and NSs in Animal and Cell Lines

The mechanisms of autophagy in particle-induced toxicity are complex, given the different physicochemical and biochemical properties of particles and the various interactions between particles and cells. Therefore, the dispersity, size, charge and coating play important roles in toxicity [148]. Recently, many inorganic nanomaterials have been frequently used to observe inorganic nanomaterial-mediated autophagy in a variety of cell lines, such as mouse dendritic cells, U251 cells, L02 cells, LLC-PK1 cells, PC12 cells, human umbilical vein endothelial cells (HUVECs), hippocampal neurons, NCI-H460 cells, HeLa cells, RAW264.7 cells and human cerebral endothelial cells [121]. Engineered nanomaterials cause chronic inflammation in the lungs of rodents [149] and induce and/or aggravate type I allergic hypersensitivity reactions including asthma, atopic dermatitis, allergic rhinitis, food allergies [150], and immune responses [151]. In addition, magnetic nanoparticles and iron oxide nanoparticles induce inflammation [152,153], autophagy markers, such as Atg5, Atg12 and LC3 [154], carcinogenic potential [17], and human neurodegenerative diseases [155], after long-term iron oxide nanoparticle exposure [156,157]. Manganese nanoparticles activate autophagy markers, such as Beclin 1 and LC3, in dopaminergic neuronal cells [158]. Quantum dots have a potential toxicity, such as oxidative damage to DNA and proteins and changes in autophagy markers, such as p62 and LC3 [159,160,161]. Quantum dots also induce autophagy markers, such as LC3 in porcine kidney cells [162]. Graphene oxide quantum dots increase autophagy markers, p62 and LC3, in the GC-2 and TM4 cell lines (male reproductive cells in mouse) [163]. Graphene oxide cause autophagy markers, p62 and LC3, in F98 rat glioblastoma cells [164]. Metal nanoparticles and carbon nanotubes (CNTs) not only affect asthma in animal models but also induce allergic airway disease [165]. Metal nanomaterials have the potential to induce dermal and respiratory allergies [166]. PM2.5 induces autophagy markers, such as Beclin 1, ULK-1, and LC3 [167,168], and results in developmental toxicity in zebrafish embryos [169], autophagy-mediated cell death in human bronchial epithelium cells [170], and cardiac dysfunction [171]. PM2.5 may represent a significant risk factor for the development of Alzheimer’s disease [172]. PM2.5 also counteracts hepatic steatosis in mice fed a high-fat diet by stimulating hepatic autophagy markers, such as p62 and LC3 [173]. PM2.5 exposure activates the autophagy markers in the spleen of SD rats, such as ATG5, VSP34, Beclin 1, and LC3 [174]. PM2.5 exposure induces renal injury and changes autophagy markers, such as p62, Beclin 1, and LC3, in rats and HK-2 cells [175]. Diesel exhaust particles (DEP) induce the generation of ROS, pro-inflammation, and apoptosis in the HUVEC tube cells [176]. DEP induce macrophage activation and dysfunction [177]. Exposure to a high-intensity traffic area affects metabolism and hormones [178]. Traffic-related PM induces autophagy markers, such as p62, Beclin 1, and LC3, in HK-2 human kidney tubular epithelial cells and rat kidney tissues [179]. In addition, silica submicrospheres [180] or zinc oxide (ZnO) nanoparticles [181] induce autophagy marker, such as LC3. ZnO nanoparticles cause the formation of ROS and autophagy marker in human ovarian cancer cells (SKOV3) [182], human epidermal keratinocytes [183], and immune cells [184]. ZnO nanoparticles result in autophagosome accumulation and autophagic cell death in PC12 cells (pheochromocytoma cells in rat) [185]. ZnO nanoparticles induce the autophagy marker, LC3, and apoptosis marker caspase-3/7 activity and change the glutathione peroxidase, superoxide dismutase, tumor necrosis factor (TNF-α), and interleukin-6 in primary astrocyte cultures [186]. ZnO nanoparticles cause the expression of annexin V, caspase-3/7 activity, and mitochondrial membrane potential, which are mediated by lipoxygenase (LOX) in human dopaminergic neuroblastoma SH-SY5Y cells [187].

Silica nanoparticles induce cardiac dysfunction in rat hearts and human cardiomyocytes [188] and cardiotoxicity in adult rat cardiomyocytes [189]. Silica nanoparticles disturb ion channels and transmembrane potentials in cardiomyocytes and induce arrhythmias in adult male C57BL/6J mice [190]. The 20 nm silica nanoparticles significantly induce apoptosis and necrosis in human endothelial cells (ECs) [191]. Silica nanomaterials induce calcium mobilization and the formation of ROS in HUVECs and adult female Balb/c mice [192]. Silica nanoparticles also increase autophagy markers, such as LC3, and autophagic cell death in HepG2 cells (human liver cancer cells) [193]. Ultrafine silicon dioxide nanoparticles trigger apoptosis in lung epithelial cells [194]. Silica nanoparticles induce inflammation in the lungs of mice [195] and the autophagy marker, p62 [196]. Amorphous silica nanoparticles cause autophagy markers, such as p62 and LC3, and vascular endothelial cell injury [197]. Silver nanoparticles increase the formation of ROS, oxidative stress [198] and the genotoxicity in human TK6 cells (lymphoblast cells) [199]. Silver nanoparticle-induced autophagy markers, such as LC3, disrupts inflammasome activation in HepG2 cells [200]. Silver nanoparticles increase autophagy markers, such as p62 and LC3, decrease the expression of transcription factors in A549 human lung adenocarcinoma cells [201], and induce other autophagy markers, such as Beclin 1 and LC3, in the adult rat brain [202]. Amine-modified silver nanoparticles trigger autophagy markers, such as P62 and LC3, and lysosomal dysfunction in NIH 3T3 cells (mouse embryonic fibroblast cells) [203]. The spleen can capture nanoparticles in Wistar rats [204]. Nanoparticles are mainly ingested by liver Kupffer cells, but splenic macrophages also play an important role [205]. Bismuth nanoparticles induce autophagy markers, such as LC3, Beclin 1, and Atg12, resulting in nephrotoxicity in the human embryonic kidney 293 cell line and kidney of BALB/c mice [206]. Bismuth nanoparticles also induce oxidative stress, such as GSH, SOD, and catalase, and apoptosis in MCF-7 cells (human breast carcinoma cells) [207]. Bismuth sulfide nanoparticles inhibit the migration and invasion in HepG2 cells and induce autophagy markers, such as p62 [208]. Bismuth nanoparticles affect the autophagy-associated cytotoxicity and cellular uptake mechanisms in human kidney cells [209]. Nanosized titanium dioxide (Nano TiO_2_) results in a potential reproduction toxicity in rat Sertoli cells (SCs), induces apoptosis, decreases cell viability, and impairs morphological structures of SCs via the related wingless MMTV integration site (Wnt) pathway [210]. Long-term exposure to nano-TiO_2_ results in liver inflammation and hepatic fibrosis in mice [211]. Nasal instillation to nano-TiO_2_ induces lung injury in mice [212]. Nano-TiO_2_ results in inflammation and fibration in mice kidneys [213]. Nano-TiO_2_ changes autophagy markers, such as Beclin 1, p62 and LC3, in podocytes [214]. Nano-TiO_2_ causes the autophagy marker, LC3, to increase in human HaCaT cells at non-cytotoxic levels [215]. Nano-TiO_2_ induces autophagic response in HeLa cells [216]. Nano-TiO_2_ induces proteostasis disruption and autophagy markers, such as LC3 and p62, in HTR-8/SVneo cells [217]. Planetary micro- and nanosized particles cause nervous system injury [218]. Copper oxide nanoparticles induce an autophagy-related response in A549 cells [219]. Copper-palladium alloy tetrapod nanoparticles induce autophagy [220]. In addition, a workplace was assessed in terms of the exposure to engineered nanoparticles of alumina, amorphous silica, and ceria used in semiconductor device fabrication [221]. One study shows workers occupational exposure to engineered nanomaterials closed to micro-sized agglomerated NSs [222]. Autophagy induces cell survival, which may induce inflammation, toxicity, and diseases.

## 4. Autophagy-Related Responses in Undecomposed MSs and NSs

**Figure 3:**
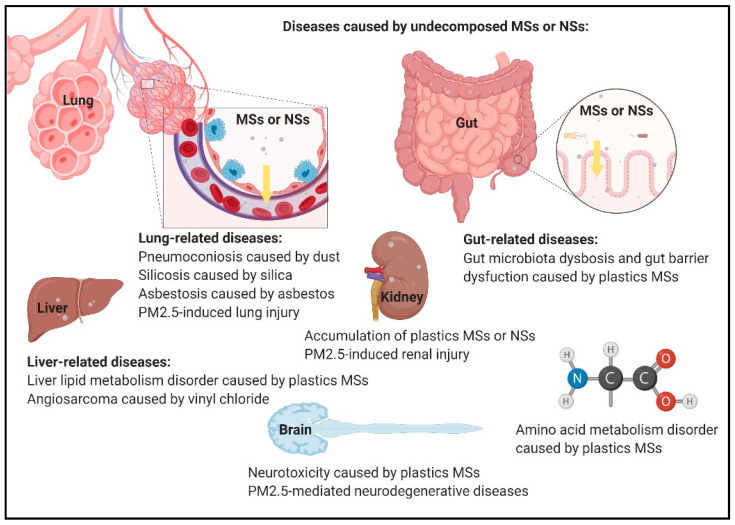
Schematic picture of undecomposed MSs and NSs causing diseases. MSs and NSs can cause obstruction, inflammation, and accumulation in organs. MSs and NSs, such as dust, silica, asbestos, plastics MSs, and PM2.5, have been found to be related to diseases in previous studies.

Briefly, plastic particles can be classified into the following three types: macroplastics (over 5 mm in size) [223], small plastic particles (less than 5 mm in size) named microplastics [224], and nanoplastics (less than 1000 nm or 100 nm in size) [225]. Recently, we overused plastic-related products. When waste plastic is fragmented into micro and nanoparticles, it can cause obstruction, inflammation, and accumulation in organs [226,227]. PS microplastics change gut microbiota dysbiosis and decrease gut mucin secretion in mice [228]. Due to their neuron toxicity, PS microplastics change the acetylcholinesterase activity in mice [135]. PS nanoplastics induce ER stress-mediated autophagy markers, such as LC3, in human lung cells [229], LGG-1, an ortholog of Atg8 on the nematode, *Caenorhabditis elegans* [230], and the autophagic marker, LC3B, in mouse embryonic fibroblasts [231]. Positively charged PS nanospheres induce autophagy markers, such as p62, Beclin 1, and LC3, in mice macrophage-like cells, RAW 264.7, and human lung epithelial cells, BEAS-2B [232]. Vinyl chloride (VC) or PVC is considered a carcinogenic factor that causes angiosarcoma in the liver [233]. VC induces fibrosis and autophagy markers, such as Beclin 1, and LC3, in kidney cells [234]. Synthetic textile workers are potentially exposed to high concentrations of microplastics in the air and suffer higher rates of lung-cancer-related mortality [235]. In addition, MSs and NSs, such as dust, silica, and asbestos, in cells are not easily decomposed. Workers exposed to high concentrations of dust are at risk of pneumoconiosis [68,236]. Pneumonoultramicroscopic silicovolcanoconiosis or silicosis is a type of pulmonary fibrosis caused by the accumulation of fine particles of crystalline silica in the lungs [237]. The prevalence of asbestosis is due to the use of asbestos-related products [238] (Figure 3). Asbestos also induces programmed necrosis in human mesothelial cells [239]. A recent study showed that asbestos induces autophagy markers, such as ATG5, p62, Beclin 1, and LC3, and mesothelial cell transformation [240]. In addition, microplastic particles were found to be deposited in urban dust [241,242,243]. Urban dust is a kind of airborne PM, containing 2–10 μm particles [244]. Recent, studies investigating MSs, NSs, and PM show that these materials may endocytose cells and result in cell death or cell survival, depending on their characteristics.

## 5. Autophagy and Tumorigenesis

We found that the previous studies show that many MSs or NSs induce autophagy (Table 1). Autophagy plays dual roles, resulting in cell death [38,245] and cell survival [246,247]. Cell survival may result in tumorigenesis [248]. Autophagy may represent a type of tumor suppressor mechanism, as it has been found that this pathway is frequently related to autophagy markers that are downregulated in tumor cells [249], which are implied to be involved in tumorigenesis [250]. Studies have indicated that a loss of autophagy function initiates cancer [251]. Autophagy is as a tumor suppressor. For example, a study indicated that mice with a deletion of atg5 and atg7 had benign liver adenomas [252]. Beclin 1 is deleted in most cases of human breast, prostate, and ovarian cancer [253]. The frameshift mutation in the ultraviolet radiation resistance-associated gene (UVRAG) decreases autophagy in colon and gastric cancers [254]. There are other proteins involved in autophagy, such as Atg4c [255], Bax-interacting factor-1 (Bif-1) [256], BH3-only proteins [257], DAP kinase [258], and PTEN [259], which shows its potential role in tumor suppression. Recently, a study showed that autophagy is involved in tumor suppression via three mechanisms. First, autophagy plays a role in tumor suppression by inhibiting necrosis-mediated inflammation. Second, autophagy plays a role in tumor suppression by maintaining genome integrity. Third, autophagy plays a role in tumor suppression by maintaining autophagy-mediated cell death and senescence [260]. In addition, autophagy plays a dual role in cancer [261]. In the beginning of tumorigenesis, autophagy prevents mutations and genotoxicity in healthy tissues due to the production of ROS [262]. However, autophagy can also be useful for tumor survival if carcinogenesis has already begun. Autophagy also helps cancer stem cells to survive stressors [263], such as cancer cell survival or chemoresistance [264]. In fact, some MSs or NSs have carcinogenic potential such as iron oxide nanoparticles [17]. PM2.5 is associated with chronic airway inflammatory diseases and lung cancer [32]. VC is considered a carcinogenic factor [233]. Asbestos causes laryngeal cancer [265]. Many MSs and NSs have been found to induce autophagy (Table 1), implying that these cells have a chance of undergoing tumorigenesis.

## 6. Solutions for MS- and NS-Caused Pollution

Many MSs and NSs may pose a potential risk to human health. How can these MSs and NSs be decreased and prevented from flowing into natural systems? Recently, some MSs and NSs have been applied in wastewater purification, such as activated carbon, carbon nanotubes, graphene, manganese oxide, zinc oxide, titanium oxide, magnesium oxide, and ferric oxides, which can be applied to remove heavy metals from wastewater [266]. In addition, wastewater treatment plants in several countries have found microplastic particles [267,268], such as the USA [268], Canada [269], and Turkey [270]. Several approaches can be used to decrease the volume of micro- and nanoplastics in water and wastewater, such as density separation, coagulation, membrane bioreactors, and biodegradation [271,272]. In addition, new techniques have been developed for water purification, such as three-dimensional graphene-based hybrid materials [273], the removal of heavy metals [274], and microplastic removal [275]. Biodegradation also seems to be a good approach, as plastic particles can be completely transformed into CO_2_ and water. Studies investigating several potential candidate marine bacteria have found that these bacteria can be used in the degradation of plastic particles [276]. Some fungal strains have been shown to degrade several plastics, such as PHB and PLA [103]. PS is known to be biodegraded in the gut of yellow mealworms because there are special microorganisms in the gut [277]. In addition, many enzymes purified from different bacteria, such as *Ideonella sakaiensis* 201-F6, have been identified and can degrade PET plastics [278]. In April 2020, a total of 436 species reported in 1451 publications were found to degrade plastic. The three types of species that can degrade plastic that were reported most often reported among the 66 different types are *Bacillus pumilus*, *Aspergillus fumigatus*, and *Phanerochaete chrysosporium*, which were found to degrade 14, 11, and 10 different types of plastic, respectively [279]. Furthermore, many enzymes have been found that can hydrolyze polyesters, such lipase, esterase, protease, cutinase, PHA depolymerase, catalase, urease and glucosidases [280]. On other hand, polyester-based biodegradable plastics, such as PLA (poly(lactic acid)), PCL (polylcaprolactone), PHB (polyhydroxybutyrate)/PHBV (Polyhydroxybutyrate-co-valarate), PBST(Poly(butylene succinate co-terephthalate), PBAT (Poly(butyrate adipate co-terephthalate)), PU (Polyurethanes) and PET (poly(ethylene terephthalate)), have potential in relation to waste reduction [280,281]. In addition, changing consumer behavior is another way to reduce plastics, such as plastic bag fee changes in Turkey [282]. The plastic carrier bag tax in Portugal reduced plastic bag consumption by 74% and increased reusable plastic bag consumption by 61% [283]. Several countries, such as the USA [284] and Caribbean countries [285], have adopted several methods for reducing single-use plastic bags.

## 7. Conclusions

Recently, EVs have played an important role in cell communication. The size of EVs, MSs, and NSs is similar. Some products made by humans are not easily decomposed. These products become environmental pollutants and bioaccumulate when they are fragmented into MSs and NSs. Among these particles, their interaction with other toxic matter has been well studied in PM2.5, MSs, and NSs. These studies have shown that MSs and NSs accumulate in organs via the food chain. In addition, MSs and NSs engulf cells and induce several cell responses, depending on their size and carrying capacity. Autophagy is a mechanism by which foreign matter decomposes in tissues or organisms. Some artificial materials are not easily decomposed by autophagy. Many MSs and NSs induce the formation of ROS, autophagic responses and apoptosis in various cells or tissues. Studies have indicated that autophagy operates through two pathways (cell death and cell survival) in tumorigenesis. MS- and NS-expressed autophagy may lead to tumorigenesis. Therefore, we found that pneumoconiosis, silicosis, and asbestosis from dust, silica, and asbestos have long disease histories, implying that MSs and NSs have previously interfered with cells and tissues and may interfere with our health through different materials in the future. Finally, the number of species of environmental bacteria and fungi found to degrade plastic seems to be increasing.

## Figures and Tables

**Table 1:** MSs & NSs of autophagy-related responses in cells or tissues.

MSs & NSs	Autophagy-Related Responses or Increasing Markers	Cells or Tissues	Reference
Magnetic nanoparticles	Autophagy markers: Atg5, Atg12, and LC3	In vitro: Human lung adenocarcinoma cells (A549) and human lung fibroblast cells (IMR-90)	[154]
Manganese nanoparticles	Autophagy markers: Beclin 1, and LC3	In vitro: Rat mesencephalic dopaminergic cells (N27)	[158]
Quantum dots	Autophagy markers: p62 and LC3	In vitro: Rat adrenal medulla pheochromocytoma cells (PC12)	[161]
	Autophagy marker: LC3	In vitro: Porcine renal proximal cell line (LLC-PK1)	[162]
Graphene oxide quantum dots	Autophagy markers: p62 and LC3	In vitro: Mouse reproductive cells (GC-2 and TM4 cells)	[163]
Graphene oxide	Autophagy markers: p62 and LC3	In vitro: Rat glioblastoma cells (F98)	[164]
Particulate matter 2.5 (PM2.5)	Autophagy markers: Beclin 1, ULK-1, and LC3	In vitro: Human bronchial epithelial cells (BEAS-2B)	[167]
	Autophagy markers: Beclin 1, ATG5,ULK-1, and LC3	In vitro: Monocytic leukemia cells (THP-1)	[168]
	Autophagy-mediated cell death	In vitro: Human bronchial epithelium cells (BEAS-2B)	[170]
	Autophagy markers: p62 and LC3	In vivo: Liver of C57BL/6 mice	[173]
	Autophagy markers: ATG5, VSP34, Beclin 1, and LC3	In vivo: Spleen of Sprague Dawley (SD) rats	[174]
	Autophagy markers: p62, Beclin 1, and LC3	In vitro: Human kidney tubular epithelial cells (HK-2)	[175]
		In vivo: Kidney of SD rat	
Diesel exhaust particles (DEP)	Autophagy markers: p62, Beclin 1, and LC3	In vitro: Human kidney tubular epithelial cells (HK-2)	[179]
		In vivo: Kidney of SD rat	
Zinc oxide (ZnO) nanoparticles	Autophagy markers: p62 and LC3	In vitro: Human cervical cancer cells (HeLa cells)	[180]
	Autophagy marker: LC3		[181]
	Autophagy marker: LC3	In vitro: Human ovarian cancer cells (SKOV3)	[182]
	Autophagy markers: p62 and LC3	In vitro: Human epidermal keratinocytes (HEKn)	[183]
	Autophagy marker LC3A and autophagic cell death	In vitro: Human T lymphoblast cells (SupT1 and Jurkat cells), C57BL/6 mouse primary splenocytes and primary human T-cells	[184]
	Autophagic cell death	In vitro: Rat adrenal medulla pheochromocytoma cells (PC12 cells)	[185]
	Autophagy marker: LC3	In vitro: Primary murine astrocytes	[186]
Silica nanoparticles	Autophagy marker: LC3 and autophagic cell death	In vitro: Human liver cancer cells (HepG2 cells)	[193]
	Autophagy marker: P62	In vitro: Human bronchial epithelial cells (BEAS-2B)	[196]
		In vivo: Lung of Bltw:CD1 (ICR) mice	
	Autophagy markers: p62 and LC3	Human umbilical vein endothelial cells (HUVECs)	[197]
Silver nanoparticles	Autophagy markers: LC3	In vitro: Human liver cancer cells (HepG2 cells)	[200]
	Autophagy markers: p62 and LC3	In vitro: Human lung adenocarcinoma cells (A549)	[201]
	Autophagy markers: Beclin 1 and LC3	In vivo: Adult brain of Wistar rat	[202]
	Autophagy markers: P62 and LC3	In vitro: Mouse embryonic fibroblast cells (NIH 3T3 cells)	[203]
Bismuth nanoparticles	Autophagy markers: Atg12, Beclin 1, and LC3	In vitro: Human embryonic kidney 293 cells (HEK293)	[204]
		In vivo: Kidney of BALB/c mice	
	Autophagy marker: p62	In vitro: Human liver cancer cells (HepG2 cells)	[208]
	Autophagy associated cytotoxicity	In vitro: Human embryonic kidney 293 cells (HEK293)	[209]
Nanosized titanium dioxide(Nano TiO_2_)	Autophagy markers: Beclin 1, p62, and LC3	In vitro: Mouse podocyte cells (MPCs)	[214]
	Autophagy marker: LC3	In vitro: Human keratinocytes (HaCaT cells)	[215]
	Autophagy marker: LC3	In vitro: Human cervical cancer cells (HeLa cells)	[216]
	Autophagy markers: p62, LC3 and autophagic cell death	In vitro: Human trophoblast cells (HTR-8/SVneo cells)	[217]
Copper oxide nanoparticles	Autophagic cell death	In vitro: Human lung adenocarcinoma cells (A549)	[219]
Polystyrene (PS) nanoplastics	Endoplasmic Reticulum(ER) stress-mediated autophagy marker: LC3	In vitro: Human bronchial epithelial cells (BEAS-2B)	[229]
	Autophagic marker: LC3B	In vitro: Mouse embryonic fibroblasts (MEFs)	[231]
	Autophagy markers: p62, Beclin 1, and LC3	In vitro: Mouse macrophage-like cells (RAW 264.7) and human bronchial epithelial cells (BEAS-2B)	[232]
Vinyl chloride (VC)	Autophagy markers: Beclin 1 and LC3	In vitro: Human kidney tubular epithelial cells (HK-2)	[234]
		In vivo: Kidney of C57BL/6 mice	
Asbestos	Autophagy markers: ATG5, p62, Beclin 1, and LC3	In vitro: Primary human mesothelial cells (HM)	[240]

## Data Availability

Not applicable.
